# Content validation of the Well-being of Older People measure (WOOP)

**DOI:** 10.1186/s12955-021-01834-5

**Published:** 2021-08-21

**Authors:** Mariska Q. N. Hackert, Job van Exel, Werner B. F. Brouwer

**Affiliations:** 1 Erasmus School of Health Policy & Management, Erasmus University Rotterdam, Burgemeester Oudlaan 50, P.O. Box 1738, 3000 DR Rotterdam, The Netherlands; 2 Erasmus School of Economics, Erasmus University Rotterdam, Rotterdam, The Netherlands

**Keywords:** Well-being, Measurement, Content validity, Older people, The Netherlands

## Abstract

**Background:**

Valid measures of the well-being of older people are important for the evaluation of health and social care services. The nine-item Well-being of Older People measure (WOOP) was based on a novel framework derived from a recent Q-methodology study, and was developed to capture a comprehensive set of well-being domains relevant to older people, as identified by themselves. This study introduces the WOOP and describes the qualitative assessment of its feasibility and content validity.

**Methods:**

Between December 2017 and January 2018, a sampling agency retrieved data from 269 adults aged 65 years and older in the Netherlands. Using an online survey, participants were asked to complete the WOOP and to indicate the importance of each item to their well-being. Open-ended questions were used to collect information about participants’ own definition of well-being, their interpretation of the items of the WOOP, and their assessment of the descriptions and response options provided with each item. Data were analysed using inductive content analysis with the software package ATLAS.ti.

**Results:**

The WOOP closely resembled respondents’ own description of what well-being means to them. The majority of the respondents reported no important well-being aspects to be missing from the WOOP, and indicated all WOOP items to be at least ‘reasonably important’ to their well-being. Many linked the WOOP items to well-being aspects as intended, and only a few had suggestions for improving the items’ descriptions and response options.

**Conclusions:**

Given these results, all nine items were retained, and no items were added to the measure. Based on respondents’ feedback, minor changes were made to the wording of some descriptions and response options of items. Concluding, the feasibility and content validity of the WOOP seem satisfactory. Further validation of this new measure is required, in different health and social care settings and among subgroups of older people with potentially different views on what constitutes well-being.

## Background

Because of the ageing of the population in many countries, the demand for care for people aged 65 years and older is growing considerably. This concerns care that improves older people’s health, but also public health, social care and long-term care services that improve their broader well-being [1]. Given that resources are limited and pressure on budgets for health care is growing, outcome measures are required that enable meaningful (economic) evaluations regarding reimbursement of these services. Traditionally, economic evaluations in the health care sector have used quality-adjusted life-years (QALYs) as outcome measure in assessing whether care services provide value for money. QALYs comprise health benefits in terms of improvements in people’s length and health-related quality of life (HRQoL). However, common HRQoL-measures were primarily developed for the evaluation of services which aim to improve health. Consequently, they may fall short in assessing the full benefits of care services for older people, as improving health is often not their primary or only aim. Well-being measures may be better able to capture the full benefits of care services for older people, as they intend to capture health and quality of life dimensions beyond health. At the moment, well-being measures are recommended to be used next to QALYs when appropriate [2]; however, they may well become the preferred outcome measures in (economic) evaluations [3]. Hence, it is necessary to gain experience with appropriate well-being measures, to enable an effective, efficient and fair allocation of scarce resources [4–6].

A number of well-being measures were developed over the years (for an overview, see [5–7]). However, most of these measures are considered less suitable to be applied in economic evaluations of care services for older people. They often lack utility scores that reflect the relative importance of domains to overall well-being [5], or they are too lengthy [8]. Short measures are better suited for the elicitation of utility scores [5], and they are more feasible in the context of self-completion by older people themselves. Also, outcome measures are often based on expert opinion, which implies that their content is not directly based on what is relevant to older people, according to themselves [8, 9]. Even if they are based on lay perspectives, heterogeneity in older people’s views on well-being is often overlooked [10]. To date, the Adult Social Care Outcomes Toolkit (ASCOT) [11] and the ICEpop CAPability measure for Older people (ICECAP-O) [12, 13] seem to be the most promising measures for use in economic evaluations of care services for older people [5, 6].

However, some recent studies have argued that both the ASCOT and the ICECAP-O may not adequately cover all domains relevant to older people’s well-being. The ASCOT was designed for use in the evaluation of social care interventions and includes domains specifically relevant for that context [11]. This limits its usefulness for the evaluation of other types of services, while using different outcome measures for different types of services reduces the comparability of outcomes across interventions and the possibility to adequately evaluate interventions that integrate different types of services. The ICECAP-O is a more generic measure, covering five broad well-being domains (such as being independent and thinking about the future without concern) in terms of capability well-being (i.e. what people *can do* or *be*). Capturing well-being through five items means that some domains that older people consider to be important are not included directly, but allegedly are captured indirectly through the measured items [12]. Important in the current context is that both the ASCOT and the ICECAP-O do not directly measure the domain health [11, 12]. While it supposedly is captured indirectly in the ICECAP-O, previous research suggests that this may not be sufficiently the case, in particular for physical health [14–17]. Still, health, and in particular physical health, was shown to be important to the well-being of older people [10, 18, 19]. Covering any relevant well-being domain indirectly may potentially lead to less attention for and impact of these domains in the evaluation and decision-making about care services [20]. Hence, it seems worthwhile to further explore outcome measures that capture all well-being domains that are important to older people more comprehensively.

This paper introduces the Well-being of Older People measure (WOOP). The WOOP is based on a novel framework derived from a recent Q-methodology study [10] that explored in-depth what a diverse sample of older people in the Netherlands consider important for their well-being. Five distinct perspectives on what constitutes well-being were found, highlighting the different domains that are important for the well-being of diverse groups of older people. This heterogeneity in views was integrated in the development of the WOOP, with the aim to arrive at a measure that captures a comprehensive set of all well-being domains relevant to all older people. This paper describes the development of the WOOP and its first qualitative validation, as crucial part of the measure’s development. In-depth information is given on tests exploring its feasibility and content validity, looking at whether all relevant well-being domains are included in the measure, whether the items and the accompanying descriptions and response options are clearly formulated, and whether respondents interpret them consistently.

## Methods

### Development of the Well-being of Older People measure (WOOP)

In the Q-methodology study [10], people aged 65 years and older in the Netherlands were presented with a set of 34 opinion statements. These statements were based on a review of the literature reporting on which aspects are relevant to older people’s well-being. In an interview-setting, respondents were asked to rank these statements, according to importance to their well-being, and to explain their ranking of the statements. Factor analysis was used to identify clusters in the ranking data. This resulted in five factors, that is, five distinct ways in which the statements were ranked by older people. These factors were each interpreted as distinct views of older people on what constitutes well-being for them, also using the rich qualitative data collected during the interviews, when older people explained their ranking of the statements. The first view emphasised the importance of health, financial security and having a life partner for pursuing all things contributing to well-being. The second view especially focused on physical functioning and the relation with family members, also in the context of receiving support when needed. The third view prioritised autonomy and helping others, and the importance of mental health as a means to this end. The fourth view emphasised the value of mental well-being, including religion, and having a support network to be able to cope with the physical frailty that comes with ageing. Finally, the fifth view emphasised the social network as well, with an emphasis on having a life partner and a pleasant living environment, and being able to adapt oneself to changing circumstances.

All in all, based on the aspects that were prioritised in the five views on what is important for well-being, the following nine domains of well-being were selected to be included in the WOOP: (i) ‘physical health’, (ii) ‘mental health’, (iii) ‘social contacts’, (iv) ‘receive support’, (v) ‘acceptance and resilience’, (vi) ‘feeling useful’, (vii) ‘independence’, (viii) ‘making ends meet’ and (ix) ‘living situation’. The first five domains emerged as important in two or more of the views on well-being. The last four domains were each considered important in one of the views particularly, highlighting the relevance of heterogeneity among older people in what constitutes well-being in the development of the WOOP.

Based on these findings, a draft version of the WOOP was developed in Dutch (see “Appendix 1” for the English translation), with one item covering each of the above-mentioned nine well-being domains. These items intend to measure functionings of older people in each of the nine domains (i.e. what people *do* or *are*). Functionings may be interpreted more straightforwardly and more uniformly than capabilities, also when older people may think quite differently about their opportunities [21, 22]. Capturing functionings also aligns conceptually with often used HRQoL-measures, which are typically based on functionings as well [5]. To ensure that the wording of the items, including their descriptions and response options, was comprehensive and clear to the target population, we used the qualitative data from the interviews in the Q-methodology study to formulate the items, descriptions and response options. Furthermore, we cross-checked the design of the items against available well-being measures in the field, including the ICECAP-O [12, 13], the ASCOT [11], the Older People’s Quality of Life questionnaire-13 (OPQOL-13) [8], the Ferrans and Powers Quality of Life Index (Ferrans and Powers QLI) [23] and the World Health Organization Quality of Life Instrument-Older Adults Module (WHOQOL-OLD) [24]. Five response options were defined for each item, to ensure relatively easy choices while keeping sufficient discriminatory power. As in this stage of the development of the WOOP utility scores are not yet available, we present a simple sum-score based on a score of 1 for the lowest level and a score of 5 for the highest level of functioning on each of the nine items, resulting in a total score ranging from 9 to 45, with higher scores reflecting higher well-being.

### Data collection

We examined this draft version of the WOOP using both a quantitative and a qualitative approach. In the quantitative approach, that has been published separately [25], a sampling agency recruited 1,113 respondents aged 65 years and older in the Netherlands, with the aim to be representative of this population in terms of age, sex and educational level. Convergent and discriminant validity, dimensionality and test–retest reliability of the WOOP were examined with satisfactory results. The focus in this paper is on the qualitative approach. Respondents that participated in the quantitative validation of the WOOP, were invited to also participate in a second online questionnaire. 269 older people agreed to this invitation, and self-completed this questionnaire at home between December 2017 and January 2018. Compared to those who participated in the first online questionnaire, respondents who also filled out this questionnaire were younger, more often male, more often retired and less often never married, and had more health problems. However, all relevant subgroups were sufficiently represented in the final sample.

The online questionnaire started with an open question about what well-being means to respondents: ‘Could you describe what well-being means to you?’. After this, they were asked to complete the WOOP, and to state whether they felt any life aspects that are important to their well-being were missing. Next, they received three follow-up questions about the items of the WOOP. Each of these questions concerned three randomly selected items from the nine items in total, ensuring that each respondent reflected on each of the nine items once. In the first question, respondents were shown only the item name (shown in bold in “Appendix 1”) and were asked to explain in their own words what that item means to them. They did this consecutively for three randomly selected items of the WOOP. In the second question, respondents were shown the full item (i.e. name, description and response options), indicating the response option they had chosen when completing the WOOP at the beginning of the questionnaire, and were asked (i) to explain why they chose that particular response option, and (ii) whether the response options belonging to that item could be improved, and if so, how this could be done. They did this consecutively for three randomly selected items of the WOOP, from the six remaining after question 1. In the third question, respondents were shown the name and description of the item and asked whether the description of the item (displayed in italics in “Appendix 1”) adequately represented the item and was clearly formulated. If not, they could indicate how it could be improved. They did this for the three remaining items of the WOOP, after question 1 and 2. After finishing this part of the questionnaire, respondents were asked to rate the importance of each item of the WOOP to their well-being and, subsequently, to the well-being of their peers (i.e. people aged 65 years and older in general), in both cases using a 7-point Likert scale ranging from ‘very unimportant’ to ‘very important’.

All questions were mandatory, which resulted in complete responses from all participants. Information about the number of older people who started but did not finish the questionnaire was not made available by the sampling agency.

### Analytic strategy

The data were analysed using inductive content analysis [26] carried out in ATLAS.ti (ATLAS.ti Scientific Software Development GmbH, Berlin, Germany). In inductive content analysis raw textual data is subjected to open coding, creating categories and abstraction through extensive examination, quantifying words and phrases, and comparison. Without using prior knowledge or theories, the aim is to move from the specific to the general, in order to systematically describe phenomena to develop context-specific meaning. First, in this study, to explore respondents’ interpretation of well-being, we open coded their answers to the question: ‘Could you describe what well-being means to you?’ into well-being aspects, which were subsequently classified into diverse well-being domains. For instance, the answer ‘good marriage’ and the answer ‘be happy with my husband’ were both coded into the aspects ‘family’ and ‘quality of contact: good’ that were later classified into the domain ‘social contacts’. Respondents were able to mention multiple aspects and, therefore, domains. However, each respondent could receive each code only once. Because we were interested in the main constituents of well-being for older people and the diversity therein, but also for reasons of clarity and conciseness, below we focus on those aspects and domains that were put forward by at least 10 out of 269 people. Respondents’ answers were coded to be missing if they did not provide a valid answer to the question. Main reasons for coding responses as missing included (i) respondents stated a synonym of well-being, and hence did not explain which aspects or domains matter for their well-being, (ii) respondents reflected on the importance of well-being to them in general, or (iii) they reported they did not know, or had an incomplete or unclear answer. The same procedure was followed when coding respondents’ interpretations of the WOOP item names. However, since each item already referred to a specific well-being domain, those responses were only coded into well-being aspects. These are presented below if they were mentioned by at least five people, as approx. 1 in 3, i.e. 90 respondents were shown each item. Regarding the issue of whether any important well-being aspects were missing, below we present those aspects that were mentioned by at least two out of 269 respondents, disregarding those aspects that were mentioned but already included in the WOOP.

We also open coded respondents’ answers to the question: ‘Could you explain whether you find (i) the description of the item and (ii) the corresponding response options clear? If not, what could be improved?’ We coded the description and response options to be clear if respondents (i) mentioned it to be ‘clear’, (ii) mentioned it to be e.g. ‘fine’ or ‘sufficient’, (iii) said ‘yes’, as we assumed that to be an answer to the first part of the question indicating it to be clear, (iv) said they ‘did not know’ or e.g. ‘nothing’ as we assumed that to be an answer to the second part of the question suggesting no need for improvements. Respondents’ answers were coded to be missing if (i) they gave no answer (i.e. only a letter or half a word), (ii) they gave an answer, but it was unclear what it meant, or (iii) they gave an answer that did not correspond to our question. For instance, some respondents reflected on their own well-being state instead of the clarity of the WOOP items. We divided suggestions for improvement into those that were WOOP item specific and those that reflected on the measure overall. We focus on issues that were put forward by at least two of approx. 90 respondents. Moreover, for each item, we used respondents’ explanation on why they had chosen a specific response option, to provide a general overview of their reasoning per item and check whether that corresponded to our intended meaning of the response options. As respondents did not use the full range of response options per item, we only provide an overview of their reasoning for the response options that were used for that item.

## Results

Table 1 displays the descriptive statistics of the study sample (for more details, see [25]). Respondents were between 65 and 94 years old, with 16% being 80 years or older. A slight majority was male and 67% was married or living together. Respectively, 39%, 34% and 27% had finished low, middle and high education. Almost all were retired. 25% indicated material deprivation in at least one of the indicated expense categories [27]. 68% reported two or more health problems on the Comorbidity index [28], 10% received informal care and 26% received at least one type of formal care.

**Table 1:** Descriptive statistics of the study sample (N = 269)

		Descriptive statistics					By age groups	
		%	Mean	SD	Min	Max	65 – 74 (N = 172)	75 + (N = 97)
Age			73	6	65	94		
Sex								
Male *(ref.* = *female)*		59					55	66
Marital status								
Married or living together		67					69	63
Never married		2					2	2
Divorced		9					11	6
Widowed		22					17	29
Education								
Low		39					37	42
Middle		34					37	30
High		27					26	28
Occupation								
Paid job		1					2	1
Volunteering		3					3	3
Retired		93					94	93
Unemployed		2					1	3
Material deprivation								
No		75					75	75
Yes, in at least one expense category		25					25	25
Comorbidity								
No		15					16	14
One		17					21	10
Two or more		68					63	75
Informal care								
No		90					90	91
Yes		10					10	9
Formal care								
No		74					82	60
Yes, use at least one type		26					18	40

Education was set to be low (primary, secondary or lower vocational education), middle (middle vocational education) and high (higher vocational or academic education). Material deprivation was defined as not being able to pay for at least one of the following four expenses: (i) heating the house, (ii) membership of a (sport)club, (iii) visiting family and friends, and (iv) paying a €1000 on unforeseen expenses without being in debt or taking a loan [27]. The number of health problems was measured using the thirteen item Comorbidity Index (CI) [28]. Informal care comprised family or friends providing care and support. Formal care concerned the use of at least one of the following services: (i) home help, (ii) home care, (iii) day-centre care, (iv) living in supported housing, or (v) living in a nursing home

238 respondents (88%) explained what well-being means to them. Most of them mentioned aspects related to health (68%) and emotional well-being (47%). Other aspects mentioned referred to social contacts (21%), finances (21%), independence (16%), or living situation (13%). A small proportion of respondents mentioned feeling useful and (health) care (both 3%, not shown). In Table 2, an overview is given of the most frequently mentioned well-being aspects, and the domains in which they were classified.

**Table 2:** Respondents’ interpretation of well-being (N = 238).

Domains	Aspects	N (%)
Health	No diseases / disabilities, (feeling) health(y)	133 (56)
	Mental health, be clearheaded, feeling mentally healthy	21 (9)
	Physical health, feeling physically healthy	17 (7)
Emotional well-being	Feeling good	48 (20)
	Feeling happy	28 (12)
	Feeling calm / stable, no stress / worries / problems	28 (12)
	Be positive / making the most of life / enjoy life	14 (6)
	Feeling content	13 (5)
Social contacts	Quality of contact: good / sufficient	26 (11)
	Family (e.g. partner, children, grandchildren)	25 (11)
	Social contacts / be socially well	16 (7)
	Friends / acquaintances	10 (4)
Finances	(Sufficient) income / financial means	21 (9)
	Ability to pay for what you want / need	19 (8)
	No financial worries / need to economize, feeling financially secure / good financial situation (prosperity)	16 (7)
Independence	Ability to do what you want / need	36 (15)
Living situation	Comfortable / nice / in harmony	16 (7)
	Neighbourhood	14 (6)

^a^Aspects that were mentioned by less than 10 respondents are not displayed

**Fig. 1:**
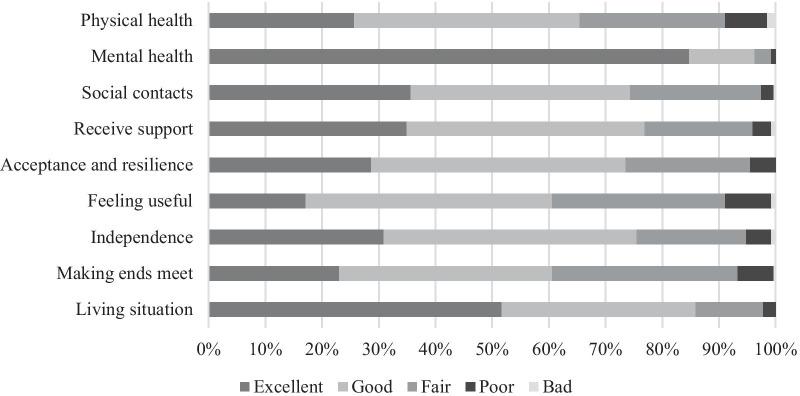
Response distribution on the items of the WOOP (N = 269). WOOP, Well-being of Older People measure. ^a^The wording of the response options differs per item (see “Appendix 1”) but follows a scale ranging from ‘excellent’ to ‘bad’ to indicate the level of well-being.

With a range of 22 to 45, the average score on the WOOP was 37. Figure 1 shows that respondents mostly reported high levels of functioning on all items. Noteworthy, almost all respondents reported an excellent or good ‘mental health’ state. The lowest levels of functioning were most frequently mentioned on the items ‘physical health’, ‘feeling useful’ and ‘making ends meet’.

**Fig. 2:**
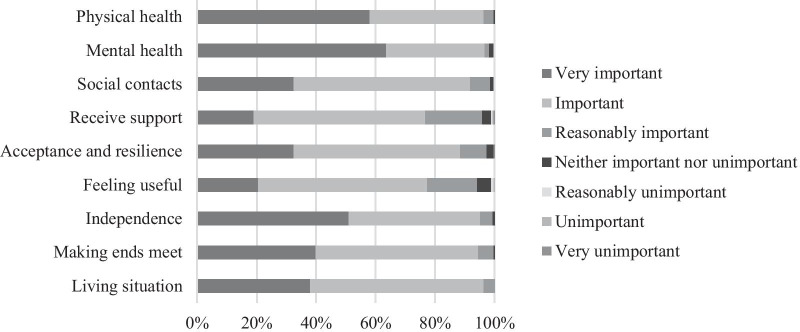
Importance of the WOOP items to the well-being of respondents themselves (N = 269). WOOP, Well-being of Older People measure

A large majority (89%) reported no important aspects of well-being to be missing from the WOOP. Those who reported aspects missing, most often referred to the well-being and health of loved ones (13% of 30 respondents), and social issues (e.g. the environment, social cohesion) (10%). Further analysis of the aspects put forward by respondents as missing in the WOOP suggested that most of these aspects were, in fact, already included in the WOOP, either directly (e.g. staying healthy) or indirectly (e.g. go on holiday). Most respondents indicated all WOOP items to be at least ‘reasonably important’ to the well-being of themselves and the well-being of their peers (see Fig. 2, responses for peers not shown). Results for these questions were very similar, with the exception that respondents more frequently chose extreme categories for themselves than for their peers (e.g. ‘very important’ versus ‘important’).

Regarding the clarity of the descriptions of the items and the response options provided to each item, several respondents reflected on their subjective nature, indicating that the interpretation of the response options could differ between people depending on their coping mechanisms and points of reference. Moreover, respondents also named item-specific suggestions for improvement, which we will discuss below.

### Physical health

87 respondents explained what ‘physical health’ means to them. Many of them linked it to their ability to do what they want or need (47%), having no diseases or disabilities or (feeling) health(y) (37%), or their mobility (24%) (see Table 3, also for other items). In general, respondents who had selected ‘no problems’ with their physical health explained this by indicating they had no diseases or disabilities, felt healthy or were able to do what they wanted. Those who reported to have ‘slight problems’ mostly indicated to have a specific health issue. Respondents opting for ‘moderate problems’ often explained this by pointing to multiple health issues, and those reporting ‘severe problems’ or ‘very severe problems’ explained this by pointing to serious limitations with mobility and functioning or constant pain. 56 respondents reflected on the clarity of the item’s description, of whom 93% had no suggestion for improvement. This was also the case for 92% of the 61 respondents who reflected on the response options. Comments made mainly reflected on the broad nature of physical health, indicating that diverse problems may be experienced differently.

**Table 3:** Respondents’ interpretation of the items of the WOOP

		N (%)
Physical health N = 87 (out of 92 respondents)		
Ability to do what you want / need		41 (47)
No diseases / disabilities, (feeling) health(y)		32 (37)
Mobility		21 (24)
Independence, no need for support (from other people)		10 (11)
No pain		8 (9)
Feeling good		7 (8)
No need for care / medicine		6 (7)
Mental health N = 78(out of 87 respondents)		
No diseases / disabilities (e.g. dementia, depression, anxiety), be clearheaded, (feeling) health(y)		36 (46)
Ability to think / understand, no confusion		19 (24)
Ability to do what you want / need		16 (21)
Feeling calm / stable, no stress / worries		12 (15)
Feeling happy / content, having a positive outlook, not feeling blue		12 (15)
Ability to deal with / accept circumstances (e.g. changes, problems)		10 (13)
Ability to remember / no forgetfulness		8 (10)
Social contacts N = 82 (out of 88 respondents)		
Family (e.g. partner, children, grandchildren)		50 (61)
Friends / acquaintances		50 (61)
Neighbours / locals		30 (37)
Other people (in general)		21 (26)
Social clubs / organisations (e.g. sport, hobbies, church)		15 (18)
Quality of contact: good / reasonable		14 (17)
Visits / meetings		11 (13)
Chat		9 (11)
Frequency of contact: regular / sufficient		9 (11)
(Previous) contacts job / volunteering		7 (9)
(Offer and receive) support		5 (6)
Receive support N = 81 (out of 88 respondents)		
Help / assistance		29 (36)
Practical help (e.g. home care, personal care)		24 (30)
Emotional / social help (e.g. empathy, friendship)		24 (30)
When needed (in general)		24 (30)
From other people (in general)		22 (27)
From family (e.g. partner, children, grandchildren)		18 (22)
With problems / setbacks / difficulties (e.g. feeling blue / lonely)		15 (19)
With diseases / limitations (also of partner)		14 (17)
From friends		6 (7)
From neighbours / locals		6 (7)
From professionals (e.g. organisations, municipality)		5 (6)
Acceptance and resilience N = 80 (out of 94 respondents)		
(Ability to) accept		33 (41)
(Ability to) deal with		27 (34)
Ageing / disabilities / limitations		27 (34)
Circumstances (past, present) / changes		24 (30)
Be content /positive / open (keep going)		22 (28)
Problems / setbacks / difficulties (e.g., loved ones passing away)		21 (26)
(Ability to) process / recover from		11 (14)
Feeling accepted / valued (by yourself / others), accepting others		9 (11)
Feeling useful N = 83 (out of 88 respondents)		
(Ability to) offer support (e.g. informal caregiving)		53 (64)
To other people (in general)		38 (46)
To family (e.g. children, grandchildren)		14 (17)
Ability to do what you want / need		13 (16)
Volunteering / working		12 (14)
(Ability to) offer practical (physical) support (e.g. home care)		8 (10)
To friends / acquaintances		7 (8)
Feeling valued / that you matter (e.g. be taken seriously)		7 (8)
Feeling useful		
(Ability to) offer emotional (social / mental) support (e.g. visits)		6 (7)
To social clubs (e.g. hobbies) / society / humankind		6 (7)
With ageing / diseases / disabilities		5 (6)
Independence N = 90 (out of 94 respondents)		
Be self-supportive, no need for support / care (from others)		64 (71)
Ability to do what you want / need		52 (58)
Be in control (e.g. thoughts, decisions, lifestyle)		19 (21)
Financial independence (e.g. having sufficient financial means)		5 (6)
Making ends meet N = 84 (out of 89 respondents)		
Ability to pay for what you want / need		52 (62)
(Sufficient) income / financial means		28 (33)
No financial problems / debts / loans		17 (20)
No financial worries / need to economize		13 (15)
Ability to save / pay for unexpected expenses		8 (10)
Ability to manage / watch your expenses		8 (10)
Living situation N = 75 (out of 87 respondents)		
House		51 (68)
Comfortable / nice / beautiful		40 (53)
Neighbourhood		25 (33)
Adjustments to age (e.g. moving, elevator, easy to maintain)		17 (23)
Interior (incl. garden / view)		7 (9)
Neighbours / locals		7 (9)
Peaceful / quiet		7 (9)
Safety		7 (9)
Living arrangements (in general)		6 (8)
(City) centre / facilities / social contacts close by		6 (8)
Affordable (e.g. rent, mortgage) / ownership		6 (8)
Spacious		5 (7)

WOOP, Well-being of Older People measure

Respondents were only shown the item name (see “Appendix 1”, shown in bold). Aspects that were mentioned by less than 5 respondents are not displayed

### Mental health

78 respondents explained what ‘mental health’ means to them, with 46% of them mentioning having no diseases or disabilities (e.g. dementia, depression, anxiety), being clearheaded or (feeling) health(y). Overall, respondents indicated to have ‘no problems’ with their mental health when they had no diseases or disabilities, felt good or had a positive outlook, and to have ‘slight problems’ or ‘moderate problems’ when they had specific health issues or struggled to deal with their circumstances. 51 respondents reflected on the clarity of the item’s description, of whom 96% had no suggestion for improvement. This was also the case for 84% of the 64 respondents who reflected on the response options. Also here, comments made reflected on the broad nature of mental health, indicating that diverse problems may be experienced differently, and more examples of different types of mental health problems were needed. In addition, some respondents commented that not all mental health problems were included as examples in the item description (e.g. being delusional).

### Social contacts

82 respondents explained what ‘social contacts’ means to them. Many of them linked it to contact with their family (e.g. partner, children, grandchildren) (61%), friends or acquaintances (61%), or neighbours and locals (37%). Mostly, respondents who reported to be ‘very satisfied’ or ‘satisfied’ with their social contacts explained this by indicating they had many, good or regular contacts, while opting for ‘reasonably satisfied’ was explained by indicating their quality of contact was poor, they had few, but good contacts or preferred to be alone. 53 respondents reflected on the clarity of the item’s description, of whom 94% had no suggestion for improvement. This was also the case for 92% of the 52 respondents who reflected on the response options. Comments made mainly reflected on the wish to consider the contact with diverse social groups separately instead of under one header.

### Receive support

81 respondents explained what ‘receive support’ means to them. Many of them associated it with receiving help or assistance (36%), practical help (e.g. home care, personal care) (30%) or emotional / social help (e.g. empathy, friendship) (30%) when needed (30%) (see Table 3). In general, respondents who reported to be ‘very satisfied’ or ‘satisfied’ with the support they received explained this by indicating they had support when needed, while opting for ‘reasonably satisfied’ was explained by indicating they needed more or better support. Those who reported to be ‘dissatisfied’ or ‘very dissatisfied’ explained this by pointing out they struggled to ask for support or felt that no one cared for them. 63 respondents reflected on the clarity of the item’s description, of whom 83% had no suggestion for improvement. This was also the case for 74% of the 65 respondents who reflected on the response options. Comments made reflected on (i) the need to make the item applicable to those who do not need to receive support, and (ii) the need to clarify which types of support need to be considered.

### Acceptance and resilience

80 respondents explained what ‘acceptance and resilience’ means to them. Most of them linked it to their ability to accept (41%) or deal with (34%) ageing, disabilities and limitations (34%). Often, respondents indicated to be ‘more than able’ or ‘able’ to deal with their circumstances when they felt good or were flexible, and to be ‘reasonably able’ or ‘barely able’ when they struggled to accept or deal with their limitations. 62 respondents reflected on the clarity of the item’s description, of whom 76% had no suggestion for improvement. This was also the case for 82% of the 57 respondents who reflected on the response options. Comments made reflected on (i) the wish to remove the reference to ‘religion and belief’ from the description of this item because it is superfluous irrespective of whether you are religious, (ii) the need to specify which circumstances and changes needed to be considered, (iii) the fact that functionings in this domain may fluctuate over time, (iv) and it may be hard to be self-aware and honest about functionings in this domain.

### Feeling useful

83 respondents explained what ‘feeling useful’ means to them, with most of them linking it to their ability to offer support (64%) to other people (46%). Mostly, respondents who reported to feel ‘very useful’ or ‘useful’ explained this by indicating they were able to support others, work or volunteer, while opting for ‘reasonably useful’ was explained by indicating they were able to do small tasks. Respondents who reported ‘do not feel useful’ or ‘do not feel at all useful’ often explained this by pointing out they were not able to perform tasks they used to do. 48 respondents reflected on the clarity of the item’s description, of whom 90% had no suggestion for improvement. This was also the case for 92% of the 61 respondents who reflected on the response options. Comments made reflected on (i) removing the reference to ‘getting appreciation’ from the description of this item because it does not reflect their motivation for being useful appropriately, and (ii) the need to specify more clearly which tasks needed to be considered.

### Independence

90 respondents explained what ‘independence’ means to them, with most of them associating it to not needing support or care from others (71%) or the ability to do what they want or need (58%). In general, respondents who reported to be ‘very independent’ or ‘independent’ explained this by indicating they felt in control or were able to do things themselves, while those who reported to be ‘reasonably independent’ or ‘dependent’ explained this by pointing out they needed (some) support. 62 respondents reflected on the clarity of the item’s description, of whom 87% had no suggestion for improvement. This was also the case for 98% of the 54 respondents who reflected on the response options. Comments made mainly reflected on the need to specify more clearly the types of independence that needed to be considered.

### Making ends meet

84 respondents explained what ‘making ends meet’ means to them. Most of them linked it to their ability to pay for what they want or need (62%), whereas some linked it to having (sufficient) income or financial means (33%) or having no financial problems, debts or loans (20%). Mostly, respondents who reported to be ‘more than able’ or ‘able’ to make ends meet explained this by indicating they were able to afford what they wanted or had no financial worries or problems. Respondents reporting to be ‘reasonably able’ to make ends meet often explained this by pointing out they needed to economise, while those reporting to be ‘barely able’ or ‘almost unable’ to make ends meet explained this by indicating they spent more than they earned and were not able to pay for what they needed. 64 respondents reflected on the clarity of the item’s description, of whom 97% had no suggestion for improvement. This was also the case for 86% of the 59 respondents who reflected on the response options. Comments made mostly reflected on the subjective nature of the item.

### Living situation

75 respondents explained what ‘living situation’ means to them, with most of them linking it to their house (68%) and, to a lesser extent, neighbourhood (33%), that both needed to be comfortable, nice or beautiful (53%). Overall, respondents who reported to be ‘very satisfied’ or ‘satisfied’ with their living situation explained this by indicating their house and neighbourhood matched their wishes, while opting for ‘reasonably satisfied’ was explained by indicating they had small defects in their house or difficulties paying for it. Those who reported to be ‘dissatisfied’ explained this by pointing out they wanted different housing or had great discomfort. 56 respondents reflected on the clarity of the item’s description, of whom 95% had no suggestion for improvement. This was also the case for 91% of the 65 respondents who reflected on the response options. Comments made mainly reflected on that scores on this item can differ between one’s house and one’s neighbourhood.

### Improvements implemented to the WOOP

Given these findings, some changes were made to the draft version of the WOOP (see “Box 1”). First, we concluded that at this stage of development of the measure no items needed to be dropped from the measure, or added to it. However, we did adjust the wording of some items. Second, although respondents generally found the descriptions of the items clear, some commented that the descriptions were not specific enough, or that specific other examples should perhaps be added to the description of certain items. This is understandable, as items were formulated at a fairly high level of abstraction to limit the number of items, but, as a consequence, sometimes aim to capture a number of underlying factors simultaneously (e.g. quantity and quality of social contacts). Rather than lengthening the descriptions in the attempt to be exhaustive, we changed the wording of the descriptions to start with ‘*consider*’ instead of the more restrictive formulation ‘*concerns*’. Third, to ensure that older people focus on their current well-being, we slightly altered the introduction text to now read: *‘*For each section, select the description *that is most appropriate for you today’*. Fourth, some adjustments were made to the wording of a number of items, descriptions and response options of the draft version of the WOOP. These changes based on the comments made by respondents, were for clarification and to improve consistent interpretation of items. The main changes are listed in “Appendix 3”. The wording of the ‘mental health’ item in Dutch was changed to *‘mentale gezondheid’* instead of ‘*geestelijke gezondheid*’, as some older people connected the original formulation to religion. We also broadened the scope of the item ‘social contacts’ by changing it to ‘social life’, potentially including consideration of the well-being and health of loved ones. Next, we altered the description of the item ‘receive support’, to make it clearer how to respond for older people who do not currently need support. The description was changed into: *Everyone needs help or support sometimes. Consider practical or emotional support, for example from your partner, family, friends, neighbours, volunteers or professionals. This concerns being able to count on support when you need it, as well as the quality of the support.’* Response options were altered in line with this, to end with *‘…the support I get, when needed’.* We also adjusted the description of the item ‘feeling useful’ in order to address the comment that quite a few respondents made about not necessarily needing appreciation to feel useful: *‘Consider meaning something to others, your environment or a good cause’.* Lastly, following these adaptations, we also made minor changes in the (order of) wording of some of the other items, in order to maintain consistency in formulation between items and to improve their comprehensibility. We did not change the description of the item ‘acceptance and resilience’, despite the suggestions of some respondents to delete the reference to religion. Reasons for not changing the description were that the study underlying the development of the WOOP [10] showed that religion is an important coping resource for many older people, and that the current wording does not seem to restrict those who are not religious in any way in answering to the item. The final version of the WOOP, including the adjustments described here above, is included in English in “Box 1” and in Dutch in “Appendix 2”. The draft and final version were developed in Dutch, and the final version was translated into English by a certified translator using the forward–backward method.

## Discussion

### Main findings

This paper introduces and describes findings concerning the feasibility and content validity of a brief, self-completion measure of the well-being of older people. The WOOP was based on a novel framework derived from a recent Q-methodology study [10], highlighting the diversity in older people’s views on well-being. The WOOP aims to capture a comprehensive set of well-being domains relevant to older people, as identified by themselves. As crucial part of the measure’s development, this paper presents in-depth information on the first qualitative validation of the WOOP. As this stage of development is often underreported, the thorough report of the techniques used in this paper may inspire others working on related instruments. Several findings of this study support that the feasibility and content validity of the WOOP seem satisfactory.

First, the WOOP items closely resembled respondents’ own descriptions of what well-being means to them. Only a few aspects that respondents mentioned as part of their well-being were not (directly) included in the measure. Most prominent in this context was (health) care, which was mentioned by 3% of respondents. We suspect that this aspect may be captured indirectly through other items included in the WOOP, such as ‘receive support’ (which also concerns health professionals). Second, 89% of the study sample reported that for them no important well-being aspects were missing from the WOOP. Those who mentioned aspects they considered to be missing, most often mentioned the well-being and health of loved ones, and social issues, but the proportion of respondents mentioning these was low. Cross-checking the wording of the WOOP items, most of the well-being aspects (often single) respondents wished to add, were in fact already (indirectly) included in the WOOP. Third, almost all respondents indicated all WOOP items to be at least reasonably important to the well-being of themselves and their peers, indicating that the measure did not include superfluous or unimportant domains. Fourth, analysis of respondents’ interpretations of the items showed that these were in line with the description given with each item, and therefore with the intended meaning of the item.

Considering the clarity of the descriptions of the items and the response options, respondents often indicated that these were clear and no improvement in the wording was necessary. Comments made by respondents indicated that they had difficulty providing an answer (i) when multiple aspects were mentioned in the description of the item, or (ii) when it was not sufficiently clear which aspects they needed to consider. Moreover, some respondents highlighted the subjective nature of the items, and that older people in different stages of ageing and in different circumstances may have different reference points, which might affect how they score their level of functioning on the various items.

## Study limitations and future research

Some limitations of this study need noting. First, quite a few responses to the questions regarding the clarity of the descriptions and response options of the WOOP items were not useful (447 out of 1,614; 28%), because respondents reflected on their well-being state (i.e. their chosen response option) rather than on the clarity of the questions and response options themselves, as requested. These responses were coded as missing. This was also observed in a few cases when respondents were asked to describe what well-being means to them, but from these responses it was often still possible to deduce what mattered to their well-being. Second, when reviewing the motivations of respondents for choosing a specific response option on an item of the WOOP, it became clear that similar motivations were given by respondents choosing different, but usually adjacent, response levels. This indicates that response levels are not absolute, but may mean different things to different people; this aligns with comments made by respondents that people may have different reference points in mind when responding to the items, for example depending on age or other circumstances. Errors and difficulties related to the response options were addressed by improving the wording of these items. A future think-aloud study may help to confirm whether the items are now interpreted (more) correctly, and more uniformly, by respondents. Also, whether the wording and layout of the WOOP can be simplified further is an interesting consideration for future research. Third, the study was designed as an online data collection, which made it possible to collect reflections on the WOOP from a sizeable and varied sample of older people. The downside of this approach is that recruitment of older people for an online survey from the panel of a sampling agency, may have resulted in a certain selection of respondents. For example, this may be the reason for the very high proportion of respondents reporting no problems with their mental health. In addition, answers to open questions in an online survey may lack the elaborateness and depth of those obtained through interviews or focus groups. Moreover, we have no information about non-response to the invitation to participate or the number of respondents that started but did not finish the questionnaire, as this information is not made available by the sampling agency. Fourth, this study included a heterogeneous sample of older people, which suits the purpose of study, but it is likely that groups of specific interest for a measure like the WOOP are not sufficiently represented. Fifth, it is possible that other researchers would have coded and interpreted the data differently. In order to minimise biases and arbitrary choices, the coding and interpretations were discussed intensively with the whole team.

### Next steps

Before the WOOP may be considered for use (next to QALYs) in (economic) evaluations of care services for older people, the results regarding its content validity, provided in this paper and the quantitative counterpart [25], should be confirmed. Further research among older people in different health and social care settings, including specific disease areas and living arrangements, but also different social and financial circumstances, socio-cultural backgrounds and the oldest age groups is required. Next, utility weights should be developed to translate different well-being profiles into a single utility score on the WOOP. Last but not least, responsiveness and sensitivity-to-change should be tested, to ensure that intervention-based changes in older people’s well-being can be captured.

## Conclusions

This study introduced and described findings concerning the feasibility and content validity of the WOOP. Based on a novel framework derived from a recent Q-methodology study, highlighting the diversity in older people’s views on well-being, the WOOP was developed to capture a comprehensive set of well-being domains relevant to older people, as identified by themselves. The qualitative data presented here also provides further insight in older people’s understanding of their well-being. Overall, the study showed favourable results regarding the comprehensiveness and clarity of the draft version of the WOOP. Some issues for improvement emerged and were incorporated in the final version of the WOOP. Future research should examine the psychometric properties of the WOOP, including its validity, reliability, responsiveness and sensitivity-to-change. Also, it is important that utility weights are determined for the instrument, so that different well-being profiles measured with the instrument can be translated into a single utility score. First tests of the construct validity and test–retest reliability conducted in parallel to the study presented here show satisfactory results. Ultimately, the WOOP will hopefully contribute to better measurement and valuation of the well-being of older people.

## Data Availability

The dataset used in this study is available from the corresponding author upon request.

## Appendix 3: Overview of the main changes to the Well-being of Older People measure (WOOP)

**Table:**

WOOP	Draft version	Reason for change	Final version
Introduction text	‘…that best fits your well-being at the moment.’	Ensure focus on current well-being	‘…that is most appropriate for you today.’
All item descriptions	‘Concerns …’	To name underlying factors as an example, without attempting to be exhaustive	‘Consider …’
Item name ‘mental health’	In Dutch ‘geestelijke gezondheid’	To loosen the original formulation from religion	In Dutch ‘mentale gezondheid’
Item name ‘social life’, response options	‘Social contacts’ ‘… about my social contacts.’	Broadening the scope of the item, including consideration of the well-being and health of loved ones	‘Social life’ ‘… with my social life.’
Item description ‘receive support’, response options	‘Concerns receiving the help and support you need, for example from your partner, family, friends, neighbours, volunteers or professionals. Consider the amount and quality of the support.’ ‘…the support I get’	To make it clearer how to respond for older people who not currently need support	‘Everyone needs help or support sometimes. Consider practical or emotional support, for example from your partner, family, friends, neighbours, volunteers or professionals. This concerns being able to count on support when you need it, as well as the quality of the support.’ ‘…the support I get, when needed’
Item description ‘feeling useful’	‘Concerns contributing or being appreciated by doing something for other people.’	People not necessarily need appreciation to feel useful	‘Consider meaning something to others, your environment or a good cause.’
Items description ‘independence’	‘… feeling free to make your own choices …’	Being able fits the context of older people better	‘… being able to make your own choices …’
Response options ‘acceptance and resilience’, ‘making ends meet’	‘I am barely able…’ ‘I am almost unable…’	To match the wording of the item ‘feeling useful’	‘I’m not able…’ ‘I’m not at all able…’

## Abbreviations

ASCOT: Adult Social Care Outcomes Toolkit
Ferrans and Powers QLI: Ferrans and Powers Quality of Life Index
HRQoL: Health-related quality of life
ICECAP-O: ICEpop CAPability measure for Older people
OPQOL-13: Older People’s Quality of Life questionnaire-13
QALYs: Quality-adjusted life-years
WHOQOL-OLD: World Health Organization Quality of Life Instrument-Older Adults Module
WOOP: Wellbeing of Older People measure
